# Psychopathology and Integrity of the Superior Longitudinal Fasciculus in Deficit and Nondeficit Schizophrenia

**DOI:** 10.3390/brainsci12020267

**Published:** 2022-02-14

**Authors:** Piotr Podwalski, Ernest Tyburski, Krzysztof Szczygieł, Krzysztof Rudkowski, Katarzyna Waszczuk, Wojciech Andrusewicz, Jolanta Kucharska-Mazur, Anna Michalczyk, Monika Mak, Katarzyna Cyranka, Błażej Misiak, Leszek Sagan, Jerzy Samochowiec

**Affiliations:** 1 Department of Psychiatry, Pomeranian Medical University, 71-460 Szczecin, Poland; kf.szczygiel@gmail.com (K.S.); krudkowski@gmail.com (K.R.); zurawska1989@gmail.com (K.W.); jola_kucharska@tlen.pl (J.K.-M.); annakarolina6@wp.pl (A.M.); samoj@pum.edu.pl (J.S.); 2 Department of Health Psychology, Pomeranian Medical University, 71-460 Szczecin, Poland; ernest.tyburski@gmail.com (E.T.); monika.mak@gmail.com (M.M.); 3 Department of Neurosurgery, Pomeranian Medical University, 71-252 Szczecin, Poland; wojciech.andrusewicz@gmail.com (W.A.); leszekm.sagan@gmail.com (L.S.); 4 Department of Psychiatry, Jagiellonian University Medical College, 31-501 Krakow, Poland; katarzyna.cyranka@gmail.com; 5 Department of Metabolic Diseases, Jagiellonian University Medical College, 31-501 Krakow, Poland; 6 Department of Psychiatry, Division of Consultation Psychiatry and Neuroscience, Wroclaw Medical University, 50-367 Wroclaw, Poland; mblazej@interia.eu

**Keywords:** white matter integrity, dti, superior longitudinal fasciculus, deficit schizophrenia, nondeficit schizophrenia, psychopathology

## Abstract

The superior longitudinal fasciculus (SLF) is a white matter bundle that connects the frontal areas with the parietal areas. As part of the visuospatial attentional network, it may be involved in the development of schizophrenia. Deficit syndrome (DS) is characterized by primary and enduring negative symptoms. The present study assessed SLF integrity in DS and nondeficit schizophrenia (NDS) patients and examined possible relationships between it and psychopathology. Twenty-six DS patients, 42 NDS patients, and 36 healthy controls (HC) underwent psychiatric evaluation and diffusion tensor imaging (DTI). After post-processing, fractional anisotropy (FA) values within the SLF were analyzed. Psychopathology was assessed with the Positive and Negative Syndrome Scale, Brief Negative Symptom Scale, and Self-evaluation of Negative Symptoms. The PANSS proxy for the deficit syndrome was used to diagnose DS. NDS patients had lower FA values than HC. DS patients had greater negative symptoms than NDS patients. After differentiating clinical groups and HC, we found no significant correlations between DTI measures and psychopathological dimensions. These results suggest that changes in SLF integrity are related to schizophrenia, and frontoparietal dysconnection plays a role in its etiopathogenesis. We confirmed that DS patients have greater negative psychopathology than NDS patients. These results are preliminary; further studies are needed.

## 1. Introduction

White matter (WM) integrity is largely responsible for the quality of communication between the various areas of the brain’s gray matter [1]. The superior longitudinal fasciculus (SLF) is a large WM bundle that connects and allows communication between the frontal, parietal, and temporal lobes [2]. There are different methods of dividing this WM structure. One of the most recent is that proposed by Nakajima et al., which is mainly based on functional communication. They distinguish the dorsal (originates in the inferior parietal lobe and terminates in the superior frontal gyrus and middle frontal gyrus), ventral (originates in the inferior parietal lobe and terminates in the middle frontal gyrus inferior frontal gyrus), and posterior parts (originates in the middle temporal gyrus and superior temporal gyrus and terminates in the inferior parietal lobe and superior parietal lobe) and the arcuate fasciculus (AF; originates in the inferior temporal gyrus, middle temporal gyrus, and superior temporal gyrus, and terminates in the posterior inferior frontal gyrus and middle frontal gyrus) [3]. Moreover, when considering the functionality and structure of the WM of the human brain, it is important to take into account the lateralization of the brain [4]. In this case, the highest level of laterality is observed in the AF of the dominant hemisphere, which is related to linguistic and cognitive functions. The main functions of the SLF are visual and spatial cognition, attention processes, control of motor processes and executive functions, and language functions [3].

Connectivity disturbances are often considered an important etiopathogenetic factor of schizophrenia [5], along with immunological dysregulation [6], intracellular metabolism [7], and genetic alterations [8]. Functional studies have identified imbalanced connectivity within the following networks: ventral attention network, thalamus network, default network, and frontoparietal network, among others [9]. WM abnormalities are often considered to be neural correlates of the communication disorder often described in schizophrenia [10]. Interestingly, the SLF, due to its anatomical connections, takes part in the abovementioned neuron networks.

WM consists mainly of axonal extensions of neurons and the surrounding glial cells. Its main function is to transmit information in the form of electrical impulses to the next nerve cells. Thanks to the development of diffusion imaging methods, it has become possible to assess the properties of WM. Diffusion tensor imaging (DTI) is a noninvasive imaging method that allows one to create a three-dimensional map of nerve bundles and has applications in psychiatric research. It uses the properties of water molecules, which in an unbounded medium undergo isotropic diffusion, while in a medium with defined limits (e.g., WM) they diffuse in an anisotropic manner. Measurement of the diffusion value, its direction, and distribution in each voxel of the imaged nervous tissue enables the reconstruction of the white matter tract [11]. The parameter most frequently used in WM research is fractional anisotropy (FA), which is a representation of the degree of ordering of water molecules diffusing in the tissue. FA values range from 0 (where the water molecule can move in any direction) to 1 (where the movement of the water molecule is limited to only one direction) [12]. Two main techniques of DTI image analysis dominate: an atlas-based approach and region of interest (ROI) analysis [13]. The ROI approach allows for a detailed analysis of specific WM bundles and thus a more detailed analysis of diffusion parameters. WM abnormalities have been widely reported in schizophrenia [14,15]. It seems that microstructural alternations of WM are in some way represented in changes in FA. The value of this index is sensitive to the various states in which the tissue is examined (e.g., myelination, axonal integrity) [16]. Disturbances of the WM tracts that interconnect cortical regions can be responsible for the production of specific symptoms. They are also connected with neurocognitive dysfunction in schizophrenia patients [17]. These changes may correlate with the duration of the disease and thus are associated with a gradual decline in functioning in this group of patients [18]. It also seems that some of these changes correlate with the symptomatology characteristic of schizophrenia. However, the results of these studies remain inconclusive despite the large number of reports [19]. This ambiguity may be due to the use of different study protocols, interpretive ambiguity, and nonheterogeneous patient populations participating in the studies [20].

A reduction in FA within the SLF has been reported in a number of studies [14,21,22,23,24]. A decrease in FA has already been reported in patients at high risk of psychosis [25,26,27,28,29,30]. However, the results are inconclusive, as Schmidt et al. described increased FA in the SLF in this population [31]. Decreased SLF integrity has been described in patients with schizophrenia, along with the disappearance of asymmetry [32]. Reports regarding the link between SLF and psychopathology are also inconclusive. The increase in FA in the SLF positively correlates with positive symptoms in the high-risk population [31]. On the other hand, schizophrenia patients are observed to have decreased FA in the SLF, which may correlate with positive symptoms [22]. Chawla et al. propose the use of reduced FA in the SLF as a marker of auditory hallucinations [33]. Interestingly, an increase in FA in motor WM tracts, including the left SLF, has been observed in patients with catatonia [34]. McClure et al. suggested that the value of FA of the SLF could be a predictor of response to therapeutic interactions such as social skills training and cognitive remediation [35].

Due to the heterogeneity of the population of people with schizophrenia, researchers wish to distinguish between different subtypes and subpopulations of patients. Hughlings Jackson provided one of the first methods to do so: dividing the symptomatology of schizophrenia into positive and negative symptoms [36]. This introduced modern dichotomous thinking about schizophrenia and facilitated the conceptualization of schizophrenia patients. In turn, the deficit syndrome (DS) was described by Carpenter et al., which is characterized by the dominance of negative symptoms in the disease symptomatology [37]. According to Galderisi et al., in order to diagnose the deficit syndrome, it is necessary to identify at least two out of a group of six symptoms. These include restricted affect, poverty of speech, curbing of interests, diminished sense of purpose, and diminished social drive. These symptoms should be persistent and last for at least 12 months. Additionally, negative symptoms are primary; that is, they do not result from side effects of treatment, depressive or anxiety symptoms, intellectual impairment, or other symptoms of psychosis [38]. The presence of the deficit syndrome determines the course of the disease, its psychopathological picture, as well as the risk factors. The premorbid functioning of patients with DS is significantly worse than that of NDS patients, especially in the early stages of life. Studies on the integrity of white matter and changes within the gray matter (GM) suggest that the etiopathogenic background of DS differs from that of NDS. Disruptions in white and grey matter are usually more pronounced in DS than in NDS and can also display a characteristic pattern [39]. Moreover, the neurocognitive functioning of these patients is disturbed [40]. The prevalence of this syndrome is estimated at 20–30% in the population of people with chronic schizophrenia [41]. Risk factors for DS include male sex, a history of schizophrenia in the family, and an increased summer birth ratio (as opposed to schizophrenia in general, where there is an increased winter birth ratio) [42]. It should be emphasized that the presence of the deficit syndrome does not exclude the presence of positive symptoms, but they do not dominate the clinical picture. Based on a variety of studies, it can be assumed that changes in white matter may constitute the endophenotype of this subpopulation of patients [43]. The neuronal background of positive symptoms in this group of patients has not been widely studied yet.

Reports of white matter changes in DS are inconclusive. The meta-analysis of Chee et al. reports that there are differences in the WM and GM of individuals with DS compared to healthy controls (HC) [44]. However, this meta-analysis did not find any difference in WM and GM between DS and nondeficit syndrome (NDS) patients. On the other hand, there are other studies showing different changes in white matter integrity between the two groups [43,45,46]. Reduction of the FA value between DS and NDS has been identified within the uncinate fasciculus [47,48,49], arcuate fasciculus [49], inferior longitudinal fasciculus [49], and the posterior part of the corpus callosum [18]. It is possible that the results of DTI analyses (together with genetic, immunological, or other neuroimaging studies) may help create potential biomarkers. The identification of patterns of neural correlates may open up the possibility of objective diagnosis in psychiatry. The construction of biomarkers may enable the stratification of patients with schizophrenia [19,50,51]. This would enable quick identification of patients with DS and, consequently, rapid intervention [39,46].

Based on the biological model of dysconnection, we hypothesize that there are differences in the SLF between individuals with deficit syndrome, nondeficit syndrome, and healthy controls. We also hypothesize that changes in SLF integrity correlate with the severity of psychopathological symptoms. The objectives of the study were formulated on this basis. The first objective was to compare FA values between the DS, NDS, and HC groups. The second was to compare psychopathology between the DS and NDS groups. The third objective was to analyze the correlation between SLF FA values and the severity of symptoms in both groups of patients with schizophrenia.

## 2. Materials and Methods

### 2.1. Participants

We recruited 68 participants from patients under the care of the Department of Psychiatry at the Pomeranian Medical University in Szczecin. Most participants were recruited from the hospital, but some patients were also recruited from outpatient care and from the day ward.

The inclusion criteria for the study included a diagnosis of chronic schizophrenia (duration of illness at least 10 years). This diagnosis was made according to the International Statistical Classification of Diseases and Related Health Problems (ICD-10) diagnostic criteria [52]. A structured questionnaire (Mini-International Neuropsychiatric Interview; MINI) was used to confirm the diagnosis [53]. Other inclusion criteria were being aged between 30 and 55 years, being able to undergo all procedures required in the project, and the ability to give informed consent to participate in the study.

The exclusion criteria included severe somatic conditions and neurological diseases, substance use disorder, and conditions that prevented the participant from undergoing the examination procedure.

The control group was composed of 36 healthy people who voluntarily agreed to participate in the study. People in this group were matched to the participants with schizophrenia in terms of age and sex. People with a history of psychiatric treatment, severe head injuries, and severe somatic or neurological diseases were excluded from the study.

All patients gave written consent to participate in the study. The study protocol was approved by the local bioethics committee.

### 2.2. Clinical Assessments

Examination of the patients was conducted in comfortable conditions. None of the patients were in acute psychosis. All patients were undergoing pharmacotherapy during the project in accordance with the current guidelines for the treatment of schizophrenia [54,55,56]. Psychopathology was assessed using the Positive and Negative Syndrome Scale (PANSS). The PANSS is a standardized tool for the multidimensional assessment of patients with schizophrenia. It consists of 30 items categorized into three groups: positive symptoms, negative symptoms, and general psychopathology. Each item is rated on a seven-point Likert scale. It is a universal tool with proven psychometric properties. [57]. To analyze the PANSS results, we used the division into five factors proposed by Shafer and Dazzi [58]. These consist of positive, negative, disorganized, affect, and resistance symptoms. For the diagnosis of DS, we used the PANSS proxy for deficit syndrome [59]. DS participants were also clinically evaluated according to the criteria proposed by Carpenter et al. [37]. The Polish versions of the Brief Negative Symptom Scale (BNSS) [60] and the Self-evaluation of Negative Symptoms (SNS) [61] were used to describe the symptoms of DS. The BNSS reliably assesses negative symptoms in five domains: anhedonia, asociality, avolition, blunted affect, and alogia, and includes an additional subscale assessing the lack of normal distress (the subscales contain a total of 13 items). The BNSS is administered during an interview with the help of additional questions contained in the manual. Each item is measured on a seven-point scale, from 0 (the lowest severity of symptoms) to 6 (the highest severity of symptoms). The SNS consists of 20 self-evaluation items. The participant places a cross in one of three boxes indicating the extent to which the situations, experiences, or feelings described applied to them over the past week. The scale has three levels: 2 (strongly agree), 1 (somewhat agree), or 0 (strongly disagree), which enables a quick assessment of negative symptoms in a patient. The examination of one participant takes approximately 5 min. Total score ranges from 0 to 40. A high score suggests a significant intensification of negative symptoms. We used the Global Assessment of Functioning (GAF) to assess overall patient functioning [62].

### 2.3. Acquisition and Measures

We acquired the DTI data with a 3.0 Tesla scanner (General Electric Signa HDxt, Milwaukee, WI, USA), using a single shot pulse sequence. The imaging parameters were diffusion-weighted, echo planar acquisition; TR = 11,675 s; TE = 82.80 ms; numbers of excitation (NEX) = 2; matrix = 96 × 96; field of view = 240 mm × 240 mm; slice thickness = 3 mm; slice gap = 0.50; acquisition time = 10 min, 19 s. Diffusion images were acquired along 25 gradient directions (b value = 1000 s/mm^2^).

### 2.4. Image Processing and Quality

We performed preprocessing, quality control, and fiber tract visualization with the ExploreDTI program. First of all, we converted DICOM files to the *.nii format, which is compatible with this software. We then checked whether the sides of the converted images matched the originals. Next, we corrected data for signal drift, removed artifacts (such as Gibbs ringing), and corrected effects due to motion and eddy current distortion. Based on this data, we created whole-brain tractography. To visualize the whole SLF, we used two regions of interest (ROIs): the first ROI was created in the association green fibers (seen superolateral to the cingulum on color map) on the coronal plane. We placed the second ROI superior to the fibers visible on the superolateral part of the cingulum at the coronal plane. Following this, we excluded parts of tracts that were not anatomically involved with the “ROInot” regions. Fractional anisotropy of the fiber tract was calculated automatically by the ExploreDTI Descriptive Statistics function [63].

### 2.5. Statistical Analysis

Statistical analysis of the results was carried out using IBM SPSS 27 (IBM Corp., Redmont, VA, USA). Continuous variables were presented as means (M) and standard deviations (SD). The normalities of the distributions were examined with the Shapiro–Wilk test, as well as the skewness and kurtosis values. We assumed that skewness values from −2 to +2 and kurtosis values from −7 to +7 indicated normal distribution of variables [64]. Age and FA parameters in the SLF were normally distributed in all three groups; negative symptoms measured with the PANSS of Kay et al. [57] were normally distributed only in the DS group; negative symptoms assessed with the BNSS and SNS were normally distributed in all three groups; years of education were not normally distributed. Chlorpromazine equivalent and global functioning on the GAF were normally distributed in both clinical groups, but psychopathological dimensions (measured with Shafer and Dazzi [58]), illness duration, and exacerbation were not normally distributed. Differences between two groups were examined with Student’s *t*-test (if the relevant assumptions were met) and the Mann–Whitney *U*-test (if the relevant assumptions were not met). Differences between the three groups were examined with the one way analysis of variance (ANOVA) *F*-test (if the relevant assumptions were met) and the Kruskal–Wallis *H*-test (if the relevant assumptions were not met). Comparisons between groups were performed using the Games–Howell or Bonferroni post hoc test (for parametric tests: ANOVA and ANCOVA, respectively) and the Dunn test (for nonparametric tests) Moreover, in the case of significant differences in FA, to control the effect of sex between the three groups and to control for chlorpromazine equivalent between the two clinical groups, we conducted an ANCOVA. Cohen’s *d* and ɳ^2^ (parametric tests) [65] or Wendt’s *r_U_*, *E*, and Cramér’s V (nonparametric tests) [49] were used to determine the magnitudes of effect sizes for differences between groups. Finally, in order to assess the relationship between the FA measures and psychopathological symptoms in both clinical groups, Pearson’s *r* and Spearman’s *rho* correlation coefficients were estimated; as there were no significant correlation coefficients, we did not conduct regression analysis. Holm–Bonferroni *p*-value correction was used for all statistical analyses (multiple comparisons and correlations). The alpha criterion level was set at 0.05 and all statistical analyses had a statistical power greater than 0.80 [66].

## 3. Results

### 3.1. Characteristics of Participants

Statistical analysis did not show significant group differences in age but there were significant differences in years of education (*p* = 0.010). Patients with DS had fewer years of education than other groups (*p* = 0.008). Moreover, there were significantly more males than females in the group of patients with DS (*p* < 0.05). After Holm–Bonferroni *p*-value correction, the clinical groups did not differ significantly in type of antipsychotic medications, chlorpromazine equivalent, duration of illness, exacerbation, or global functioning measured by GAF. Moreover, patients with DS had greater severity of negative symptoms than patients with NDS measured with the PANSS of Shafer and Dazzi [58] (*p* < 0.001) and negative symptoms measured with the PANSS of Kay et al. [57] (*p* < 0.001), and negative symptoms assessed by two additional scales: BNSS (*p* < 0.001) and SNS (*p* < 0.001). After Holm–Bonferroni *p*-value correction, there were no significant differences between the clinical groups in the severity of the other psychopathological dimensions measured by PANSS. All demographic and clinical characteristics are presented in Table 1.

### 3.2. Differences in FA Measures

**Figure 1:**
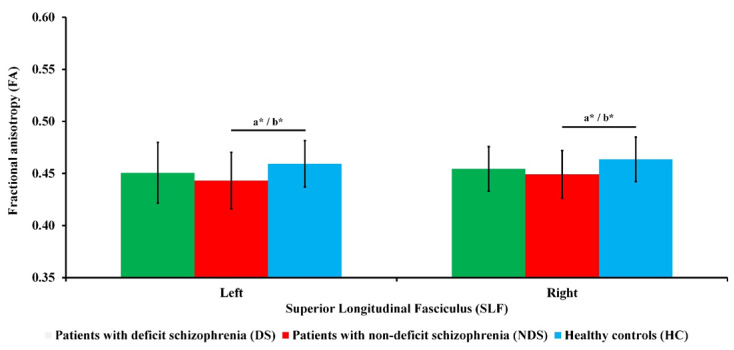
Fractional anisotropy (FA) of the superior longitudinal fasciculus (SLF) for all groups. Standard deviations (*SD*) are presented as bars. ^a^ Significant difference for ANOVA post hoc. ^b^ Significant difference after covarying sex for ANCOVA post hoc. * *p* < 0.05.

As can be seen in Figure 1, there were significant differences in FA in the left SLF (*F*_(2, 101)_ = 3.68; *p* = 0.029; ɳ^2^ = 0.07) and FA in the right SLF (*F*_(2, 101)_ = 4.17; *p* = 0.018; ɳ^2^ = 0.08) between the three groups. Post hoc analysis showed that patients with NDS had lower FA in the left SLF than HC (*p* = 0.014) and in the right SLF (*p* = 0.015) than HC. The differences in FA in the left SLF (*F*_(2, 100)_ = 4.04; *p* = 0.021; ɳ^2^ = 0.08) and FA in the right SLF (*F*_(2, 100)_ = 4.90; *p* = 0.009; ɳ^2^ = 0.09) between the three groups after adjusting for sex remained significant. Pairwise comparisons showed that patients with NDS had lower FA in the left SLF (*p* = 0.018) and in the right SLF (*p* = 0.010) than HC. There were no significant differences in FA between the two clinical groups, even after adjusting for the possible impact of medication (chlorpromazine equivalent).

### 3.3. Relationship between FA Measures and Psychopathological Dimensions

Statistical analysis after Holm–Bonferroni *p*-value correction did not show any significant correlations in DS or NDS patients between FA in the left or right SLF and psychopathological dimensions: positive symptoms, negative symptoms, disorganization, affect, or resistance measured with the PANSS of Shafer and Dazzi [58] or negative symptoms measured with the PANSS of Kay et al. [57], or negative symptoms assessed with BNSS and SNS.

## 4. Discussion

Using the diffusion tensor imaging (DTI) methodology, we explored the integrity of the superior longitudinal fasciculus (SLF) in the deficit syndrome (DS) and nondeficit syndrome (NDS) patient populations. We were able to detect changes in fractional anisotropy (FA) values among the NDS group in the right and left SLF compared to HC. We also confirmed the difference in psychopathology between DS and NDS in terms of negative symptoms. However, we did not find a difference between the FAs of the SLF in DS and NDS patients, nor any association between SLF integrity and the psychopathology of schizophrenia.

The global decline in WM integrity in patients with schizophrenia has been extensively described by the ENIGMA Schizophrenia Working Group in a study conducted on a population of 1963 patients with schizophrenia [13]. Decreased FA in the whole brain was also confirmed by Koshiyama et al., suggesting that WM structural abnormalities may underlie the etiopathogenesis of various mental disorders. Research in this area helps improve diagnosis, can be the basis for the creation of new diagnostic classifications, and contributes to the formation of new phenotypes of mental diseases [14]. These reports also appear to be in line with the dysconnection hypothesis [67]. The functional disconnection of the various cortical areas that Friston proposed as the etiopathogenetic basis of schizophrenia [5] has, to some extent, been confirmed by functional MRI studies [9,68]. It is possible that abnormal WM integrity is a neuronal correlate of impaired communication between cortical areas of the brain. DTI is a method that allows us to analyze the quality of WM, which gives us information about the diffusion of water in the nervous tissue. However, it is unclear what the FA value actually indicates, and this makes interpretation difficult [12]. The neurobiological mechanism underlying FA reduction is unknown. Reduction in FA is often interpreted as abnormal fiber structure, abnormal myelination, or abnormal axonal morphology. These interpretations are based on neuropathological examinations in which ultrastructural changes in myelin and changes in oligodendrocytes—density and number, but also axon atrophy—were found [69]. However, we are unable to pinpoint the exact cellular pathology using FA values. Nonetheless, FA is likely to affect connectivity. These disturbances may be related to abnormal neurodevelopment leading to excessive neuronal pruning. In effect, incorrect signal transduction may contribute to functional abnormalities and, consequently, to the development of psychopathological symptoms.

Data on SLF integrity in schizophrenia remain inconclusive. Some reports are consistent with our results, identifying a reduction in FA bilaterally in the SLF [33]. On the other hand, other researchers note that SLF integrity disorders are part of the neuroanatomical picture of impaired left-lateralization in patients with schizophrenia and have found reduced integrity on the left side of the SLF [70,71,72,73,74]. Some studies have found reduced FA within the SLF only on the right side in psychosis [22,32,75]. Interestingly, Kristensen et al. reported changes in the integrity of the right SLF already in UHR patients, which may indicate the primary nature of this phenomenon [75]. Ambiguous results are often due to the limitations of the DTI methodology, the use of a variety of research protocols and equipment, and post-processing difficulties. On the other hand, nonheterogeneous groups of research participants may also influence the results. In our study, we were unable to observe differences in SLF integrity in DS patients. According to a review by Tan et al., alteration of SLF integrity in DS patients has only been found in one study [43]. Rowland et al. reported that in patients with DS there was a reduction in the FA value of the SLF on the right side compared to healthy people [76]. Further research on this population of patients with schizophrenia is extremely important due to the poorer prognosis of DS patients and greater difficulties in treatment of negative symptoms [77]. Moreover, patient stratification may also bring us closer to the development of biomarkers in psychiatry [51].

The SLF is one of the structures included in the visuospatial attentional network, consisting of frontoparietal connections. Abnormal frontotemporal communication is regarded as a possible neural background of schizophrenia [32]. Hatton et al. reported a relationship between reduced left SLF integrity and neurocognitive disorders in the areas of sustained attention and verbal fluency in patients with early psychosis [70]. Similar relationships also exist in patients in the first episode of the disease, which emphasizes the importance of disturbances within this structure [78]. Perhaps decreased FA values in the SLF (an important component of the frontotemporal network) underlie core aspects of schizophrenia. A link has been identified between the cumulative risk of schizophrenia and FA values in the SLF [79]. Interestingly, Seok et al. demonstrated a positive correlation between auditory hallucinations in patients with schizophrenia and FA values in the left frontal part of the SLF [72]. Chwala et al. propose the use of FA values in the SLF and arcuate fasciculus as a biomarker for the presence of auditory hallucinations [33]. On the other hand, changes in SLF integrity may also correlate with negative psychopathology as measured by the PANSS scale [22]. Our study did not identify relationships of psychopathology in DS and NDS patients with FA values in the SLF. The lack of a relationship between the symptomatology and the integrity of SLF may be the result of the study group being too small. It could also be related to the preliminary nature of this study. There are few reports of the association of negative symptoms with the microstructure of white matter. Ohtani et al. found a relationship between FA reduction within the left posterior network of connections between the medial orbitofrontal cortex and the rostral part of the anterior cingulate cortex and anhedonia–asociality and avolition–apathy as measured by the Scale for the Assessment of Negative Symptoms [80]. Another study identified a correlation between positive and negative symptoms and the values of the right inferior fronto-occipital fasciculus in patients during the first episode of the disease [81]. Changes identified by Tan et al. in patients with DS within the body of the corpus callosum and right posterior thalamic radiation positively correlated with cognitive control and emotional awareness. These changes may underlie negative symptoms such as blunted affect [43]. Rowland et al. also found no relationship between symptomatology and SLF integrity in DS patients [76]. This may be related to the selection of the group: patients with low intensity of positive symptoms participated in our project to facilitate comparison of negative symptoms. Intensified positive symptoms may make the diagnosis of the deficit syndrome difficult due to the possibility of the appearance of secondary negative symptoms [37,42,77]. The lack of a relationship between negative psychopathology and the structure of WM may indicate a more complex mechanism of symptom formation [39]. It seems that the participation of neurotransmitters, alterations within a single cell or synapse, and the participation of dendritic spines may play an important role here. There is little research investigating this, and further exploration in this area is needed. The group of patients we studied also did not present any motoric disorders characteristic of catatonia. Interestingly, it turns out that FA values in the SLF may positively correlate with catatonic symptoms [34]. This report is directly related to the history of the dysconnection hypothesis. Carl Wernicke, in his description of motility psychosis, looked for a connection disorder within the “mental reflex arc”, creating the sejuction hypothesis that underlies modern thinking about dysconnection in schizophrenia [5,82].

Our study has some limitations. First of all, studies that require large financial outlays do not use large groups of respondents. Perhaps increasing the number of participants in the project would help obtain more significant results in individual groups. Moreover, in our study, we used a PANSS proxy, instead of the Schedule for the Deficit Syndrome, to classify patients as DS and NDS subjects. Another limitation is the unequal ratio of men to women in the DS group. Moreover, the very definition of the group of patients with deficit schizophrenia is not uniform across published studies, which makes the comparison of results of different studies more difficult. We would also like to note that there are many nonspecific factors of a very individual nature, such as physical activity, lifestyle, diet, and stimulants (including alcohol and nicotine), that can independently affect the assessed structures of white matter. It should be mentioned that the control group was selected based on gender and age. The study groups could be better standardized by deepening the psychiatric assessment with other psychopathological dimensions, such as depression or anxiety. This requires further research, which our team intends to undertake. We believe that in future research it will be important to take into account other DTI parameters and to expand the study groups using a range of psychopathological assessments.

## 5. Conclusions

In conclusion, our study investigated the relationship of WM integrity within the SLF bundle in DS and NDS patients. We were able to identify changes in SLF integrity in patients without DS compared to HC. We also confirmed a significant difference in psychopathology between DS and NDS in the dimension of negative symptoms. This is important from the point of view of confirming this diagnostic category. We did not detect significant changes in SLF integrity between DS and NDS participants. We also did not identify a relationship between SLF integrity and the psychopathological dimensions of schizophrenia.

## Figures and Tables

**Table 1:** Demographic and clinical characteristics of participants.

	Patients with Deficit Schizophrenia (DS) (*n* = 26)	Patients with Nondeficit Schizophrenia (NDS) (*n* = 42)	Healthy Controls (HC) (*n* = 36)	*F*/*H*/*χ^2^*/*t*/*Z*	*p*	ɳ^2^/*E*/V/*d*/*r_U_*
Age: *M* (*SD*)	38.38 (6.47)	38.61 (7.12)	37.39 (7.82)	0.30 ^a^	0.740	0.01 ^f^
Years of education: *M* (*SD*)	12.28 (2.90)	13.33 (2.58)	14.53 (2.63)	9.31 ^b^	0.010	0.09 ^g^
Sex: female/male	7/19	23/19	21/15	6.88 ^c^	0.032	0.22 h
Antipsychotic medications:						
Atypical: *n* (%)	18 (69.23)	26 (61.90)	-	2.12 ^c^	0.547	0.12 ^h^
Atypical and typical: *n* (%)	7 (26.93)	12 (28.60)	-			
Typical: *n* (%)	0 (0.00)	3 (7.10)	-			
No medications: *n* (%)	1 (3.84)	1 (2.40)	-			
Chlorpromazine equivalent (mg): *M* (*SD*)	698.73 (321.77)	627.50 (303.75)	-	0.92 ^d^	0.724	0.23 ^i^
Duration of illness: *M* (*SD*)	16.92 (6.01)	13.60 (4.90)	-	−2.31 ^e^	0.084	0.33 ^j^
Exacerbation: *M* (*SD*)	5.62 (2.53)	6.71 (5.11)	-	−0.19 ^e^	0.854	0.03 ^j^
Global functioning in GAF: *M* (*SD*)	50.40 (15.14)	58.53 (14.53)	-	−2.18 ^d^	0.099	0.54 ^i^
PANSS (by [48]):						
Positive Symptoms: *M* (*SD*)	7.46 (2.8)	8.14 (4.5)	-	−0.12 ^e^	1.000	0.02 ^j^
Negative Symptoms: *M* (*SD*)	22.81 (4.5)	13.29 (4.3)	-	−5.98 ^e^	0.000	0.87 ^j^
Disorganization: *M* (*SD*)	12.81 (3.5)	11.21 (3.8)	-	−2.24 ^e^	0.100	0.32 ^j^
Affect: *M* (*SD*)	8.58 (3.5)	9.26 (3.6)	-	−0.93 ^e^	1.000	0.13 ^j^
Resistance: *M* (*SD*)	4.38 (0.6)	4.90 (2.5)	-	−0.17 ^e^	1.000	0.02 ^j^
PANSS (by [57]):						
Negative Symptoms: *M* (*SD*)	20.77 (4.1)	12.93 (3.6)	-	−5.76 ^e^	0.000	0.83 ^j^
BNSS total score: *M* (*SD*)	47.42 (9.6)	19.48 (11.8)	-	−6.05 ^e^	0.000	0.88 ^j^
SNS total score: *M* (*SD*)	22.54 (7.8)	9.52 (6.9)	-	−5.35 ^e^	0.000	0.77 ^j^

BNSS = Brief Negative Symptom Scale. GAF = Global Assessment of Functioning. PANSS = Positive and Negative Syndrome Scale. SNS = Self-evaluation of Negative Symptoms. ^a^ One-way analysis of variance *F*-test. ^b^ Kruskal–Wallis *H*-test. ^c^ Chi-squared test. ^d^ Student’s *t*-test. ^e^ Mann–Whitney *U*-test. ^f^ Eta squared effect size: small (0.01–0.059), medium (0.06–0.139), large (0.14–1.00). ^g^ Epsilon squared effect size: small (0.10–0.29), medium (0.30–0.49), large (>0.50). ^h^ Cramér’s V effect size: small (0.20–0.49), medium (0.50–0.79), large (>0.80). ^i^ Cohen’s *d* effect size: small (0.20–0.49), medium (0.50–0.79), large (>0.80). ^j^ Wendt’s *r* rank-biserial correlation effect size: small (0.10–0.29), medium (0.30–0.49), large (>0.50).

## Data Availability

Data and materials for the experiments reported here are available from the corresponding author on reasonable request.
